# Effects of various remineralizing agents on the outcome of post-orthodontic white spot lesions (WSLs): a clinical trial

**DOI:** 10.1186/s40510-016-0138-9

**Published:** 2016-08-02

**Authors:** Sombir Singh, Satinder Pal Singh, Ashima Goyal, Ashok Kumar Utreja, Ashok Kumar Jena

**Affiliations:** 1 Unit of Orthodontics, Oral Health Sciences Centre Post Graduate Institute of Medical Education and Research, Sector-12, Chandigarh, India; 2 Unit of Pedodontics and Preventive Dentistry, Oral Health Sciences Centre Post Graduate Institute of Medical Education and Research, Sector-12, Chandigarh, India; 3 Department of Dental Surgery, All India Institute of Medical Sciences Sijua, Dumduma, Bhubaneswar, Odisha, India

**Keywords:** Orthodontics, White spot lesion, Fluoride toothpaste, Fluoride varnish, CPP-ACP crème

## Abstract

**Background:**

One of the most undesirable side effects of comprehensive orthodontic treatment is white spot lesions (WSLs). Despite many attempts at prevention of WSLs, its prevalence remains very high on debonding. There are many agents like fluoride toothpastes, fluoride varnishes, and fluoride mouth rinses, and casein phosphopeptide-amorphous calcium phosphate (CPP-ACP) is frequently used for the remineralization of WSLs. However, there is no consensus in the literature with respect to the success rates of these agents. Thus, the present study was designed to evaluate the efficacy of fluoride toothpaste alone and in combination with fluoride varnish and CPP-ACP plus crème in the remineralization of post-orthodontic WSLs.

**Methods:**

Forty-five subjects in the age range of 16–25 years having at least one post-orthodontic WSL were included in the study. All the subjects were randomly divided into three groups (toothpaste group, varnish group, and CPP-ACP group). The efficacy of various remineralizing agents on the remineralization of WSLs was evaluated clinically and by DIAGNOdent immediately after debonding and subsequently after 1, 3, and 6 months of their use.

**Results:**

Twice daily use of fluoride toothpaste alone had no significant effect on remineralization of WSLs at various intervals of observations (*P* = 0.078). Application of fluoride varnish along with twice daily use of fluoride toothpaste for 6 months significantly decreased the severity of WSLs (*P* < 0.01). Twice daily use of CPP-ACP plus crème along with fluoride toothpaste had significant effect on remineralization of WSLs at the end of 6 months of observation (*P* < 0.05). Between the group comparison showed that the mean visual and DIAGNOdent scores at various time intervals of observations were decreased more when fluoride varnish and CPP-ACP crème were used in addition to daily use of fluoride toothpaste, but the differences were not statistically significant (*P* > 0.05).

**Conclusions:**

The use of fluoride varnish and CPP-ACP plus crème in addition to twice daily use of fluoride toothpaste had no additional benefit in the remineralization of post-orthodontic WSLs.

## Background

White spot lesions (WSLs) are one of the most undesirable side effects of multibracket orthodontic treatment and have been reported to occur in up to 96 % of these patients [1, 2]. Despite many attempts at comprehensive prophylaxis aiming at prevention of WSLs, the prevalence of WSLs remain as high as 61 % on debonding [3]. It is generally believed that these lesions will recover through natural remineralization through saliva, once the orthodontic appliances have been removed and oral hygiene is restored [4]. However, natural remineralization through saliva accounts for mineral gain in the surface layer of WSLs has little improvement on the esthetics and structural properties of the deeper lesions [5]. The complete elimination of WSLs is unlikely [6], and some WSLs last for up to 5–12 years [7]. Therefore, it becomes necessary to apply remineralizing agents to repair the deeper parts of WSLs for better esthetics.

The commonly used agents for the treatment of WSLs are topical fluorides [8–11]. These include fluoride toothpastes [12–14], fluoride varnishes [15], and fluoride mouth rinses [4, 16]. Recently introduced casein phosphopeptide-amorphous calcium phosphate (CPP-ACP) is frequently used for the treatment of WSLs [17–20]. Most topical fluoride products rely on patient compliance and render some of these methods less efficacious. The topical fluoride delivery methods such as application of varnish and other remineralizing agents provide adequate control and reduce the need for patient compliance [15, 18]. However, there is no consensus in the literature with respect to the success rates of these agents. Thus, the present study was designed to evaluate the efficacy of fluoride toothpaste alone and in combination with fluoride varnish and CPP-ACP plus crème in the remineralization of post-orthodontic WSLs. The null hypothesis which was tested in the present study was that fluoride toothpaste alone and in combination with fluoride varnish and CPP-ACP crème were equally effective in the remineralization of post-orthodontic WSLs.

## Methods

The study was approved by the Institute Review Board (NK/1155/MDS/4037-38).

### Sample size calculation

Sample size of 36 was calculated, demonstrating a 20 % decreases in the severity of WSLs between the control and experimental groups, power of 80 %, level of significance as 95 %, and the estimated disease reduction in the control group as 50 %. But considering the expected loss to follow-up as 25 %, the final sample size was inflated to 45.

### Assessment for eligibility

Subjects in the age range of 16–25 years who just completed comprehensive orthodontic treatment with at least one WSL in a specific mouth area were considered as inclusion criteria, and subjects with cleft lip and palate deformities, orofacial syndrome, hypoplastic enamel defects, and multiple restorations on the facial surfaces and with known history of allergy to milk protein were considered as exclusion criteria. A written informed consent of minors’ parents was obtained from each patient.

### Enrollment

All the subjects were randomly allocated to three groups of 15 each using block randomization approach with 3 blocks, having a block size of 5 subjects each. Of all subjects recruited in the study (*n* = 45; *M* = 21, *F* = 24), 4 (*M* = 3, *F* = 1) subjects were lost during the follow-up period.

### Allocation

**Fig. 1:**
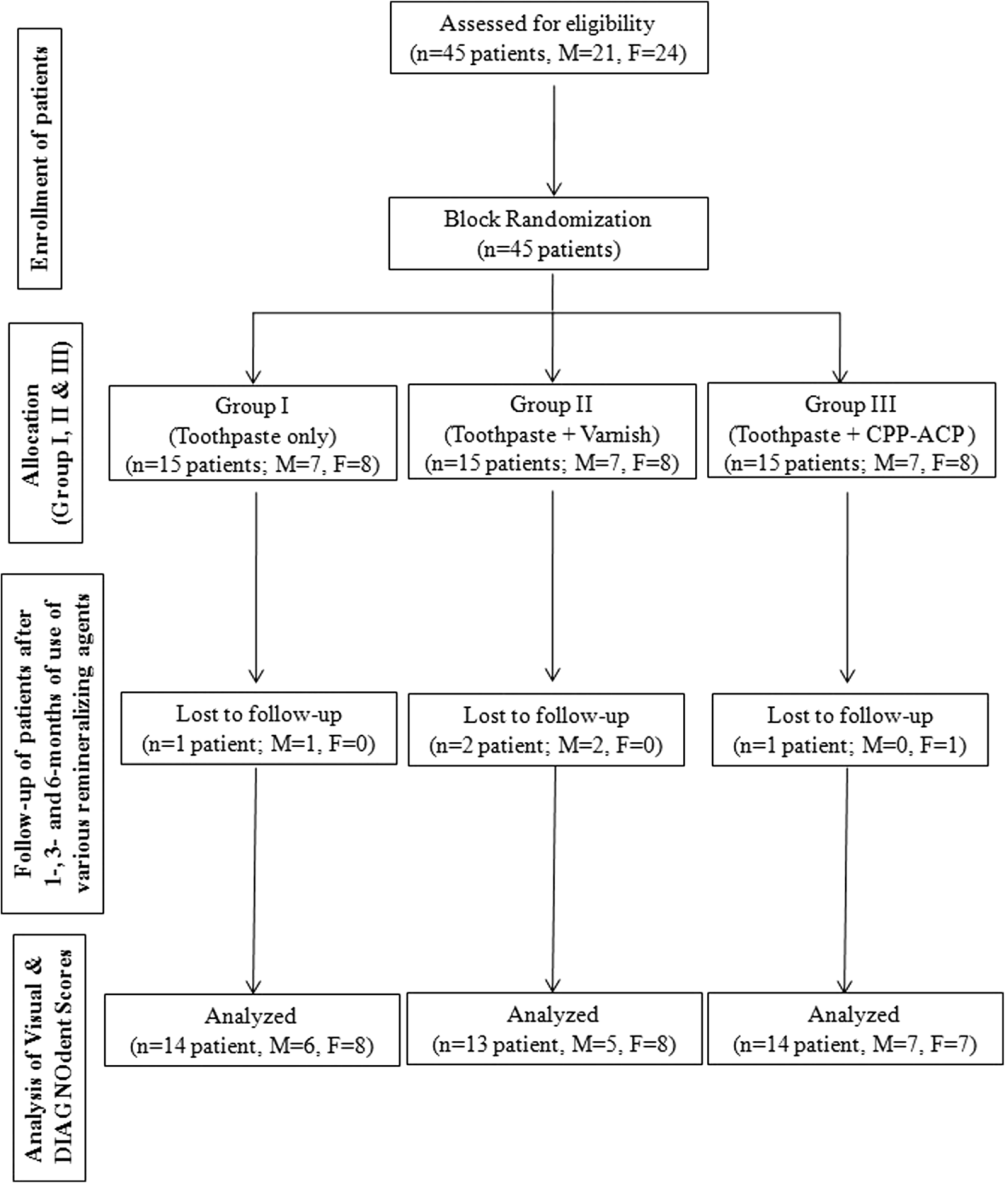
Flow chart showing patient allocation

Groups I (*n* = 14; *M* = 6, *F* = 8), II (*n* = 13; *M* = 5, *F* = 8), and III (*n* = 14; *M* = 7, *F* = 7) included subjects who used toothpaste, fluoride varnish, and CPP-ACP plus crème, respectively. A random code number were allotted to each patient at the beginning of study. The patients and the data evaluators were blinded with respect to the type of remineralizing agents used. In each subject, the efficacy of remineralizing agents was assessed both by subjective and objective methods immediately after debonding (*T*_0_), i.e., prior to application of remineralizing agents and subsequently 1 month (*T*_1_), 3 months (*T*_2_), and 6 months (*T*_3_) of use of various remineralizing agents. A participant flow chart and intervention allocation is shown in Fig. 1.

Group I (control group) subjects were advised to brush twice daily with 1000 ppm fluoride toothpaste (Colgate total® Colgate-Palmolive Company) during the follow-up period. No other fluoride supplements were allowed for use.

In group II (varnish group), fluoride varnish (5 % NaF, Fluoritop-SR® ICPA Health Products Ltd.) was applied. The labial surface of each tooth surface was polished by non-fluoridated pumice powder and was rinsed and dried thoroughly. Approximately 1 ml of varnish was applied by paint-on technique on the labial surface of each tooth in both the jaws from central incisor to the first molar on either side. After varnish application, patients were advised not to drink water for at least 30 min, eat for 4 h, and brush their teeth until the next day morning. The subjects were then advised to brush their teeth twice daily with 1000 ppm fluoride toothpaste (Colgate total® Colgate-Palmolive Company). No other fluoride supplements were allowed for use.

The group III (CPP-ACP group) subjects were advised to use pea size CPP-ACP plus crème (GC Tooth Mousse, Asia Pty. Ltd, Japan) on the tooth surfaces using a clean finger twice daily following brushing their teeth with 1000 ppm fluoride toothpaste (Colgate total® Colgate-Palmolive Company). The subjects were advised to keep the CPP-ACP plus crème over the teeth surfaces for at least 3 min before rinsing the mouth. The CPP-ACP plus crème contains 900 ppm of fluoride along with CPP-ACP crème, releases calcium and phosphate and helps in maintaining a supersaturated solution in the oral cavity. After the application of CPP-ACP plus crème, the subjects were advised not to drink water or eat anything for at least 30 min. No other fluoride supplements were allowed for use.

### Outcome measures

The WSLs were recorded on a standard proforma. The labial surface of each tooth in both the jaws from central incisor to the first molar on either side was considered in the study. The teeth were polished by non-fluoridated pumice powder and were rinsed and dried thoroughly for subjective and objective recording of WSLs. In subjective method, the scores and criteria proposed by Boyd [14] were used (Table 1). The DIAGNOdent was used to quantify the WSLs objectively. Measurements with the DIAGNOdent were performed and calibrated for each patient on a sound enamel (incisal third of the central incisor) before actual readings. The teeth were scanned carefully with the tip held in contact with the tooth surface and tilted around the measuring site so that fluorescence could be collected from all directions. The maximum reading was recorded. Each labial surface was measured thrice (by AG), and finally, a mean of all the three readings was calculated.

**Table 1:** Scores and criteria followed for the subjective evaluation of the WSLs

Scores	Criteria
0	No visible white spot or surface disruption (no decalcification)
1	Visible white spot without surface disruption (mild decalcification)
2	Visible white spot lesion having a roughened surface but not requiring restoration (moderate decalcification)
3	Visible white spot lesion requiring restoration (severe decalcification)

For each subject, the scores of all the teeth were added and the mean was considered for statistical analysis.

### Statistical analysis

A master file was made in Microsoft excel software and the data statistically analyzed on a computer using SPSS software version 17 (Statistical Packages for the Social Sciences, Chicago, IL). Descriptive statistics were used. The ANOVA and Bonferroni were used for within the group comparisons. The Kruskal-Wallis test and ANOVA were used for between the group comparisons. A *P* value of <0.05 was considered as level of statistical significance.

## Results

The mean age of the subjects at the beginning of the study was 18.93 ± 2.97, 19.08 ± 3.57, and 16.93 ± 3.24 years for group I, group II, and group III, respectively (*P* = 0.953). Total 913 teeth were examined. At the beginning of the study, a total of 556 WSLs were recorded subjectively, of which 230 and 326 were present among males and females, respectively.

The changes in the visual scores are described in Table 2. The mean visual score at the beginning of study was comparable among the three groups (*P* = 0.733). The mean scores in all groups were decreased at *T*_1_, *T*_2_, and *T*_3_ observation. In group I, there was no significant change in the mean visual scores at various time of observation (*P* = 0.078). In group II, the change in the visual scores was significant (*P* < 0.05). The visual scores in group II subjects decreased significantly at *T*_3_ compared to *T*_0_ (*P* < 0.01). In group III, the change in the visual scores was significant (*P* < 0.01). The mean visual scores in group III subjects decreased significantly at *T*_2_ (*P* < 0.05) and *T*_3_ (*P* < 0.05) compared to *T*_0_. The changes in the visual scores between the groups at various time intervals of observation (*T*_1_, *T*_2_, and *T*_3_) are described in Table 3. The mean visual scores at *T*_1_, *T*_2_, and *T*_3_ were decreased in all the groups, but the decrease in group II and group III was more compared to the decrease in group I. However, the decrease in the mean visual scores among three groups at various time intervals of observation was comparable (*P* > 0.05).

**Table 2:** Description of visual scores among subjects of three groups at various time intervals of observation

Groups	Time intervals	Visual scores mean ± SD	Significance (*P* value)	Comparison between various time intervals of observation (intra-group)	
				Comparison	*P* value
Group I	*T*_0_	15.71 ± 7.94	0.078 NS	*T*_0_–*T*_1_	1.000 NS
	*T*_1_	12.14 ± 8.41		*T*_0_–*T*_2_	0.582 NS
	*T*_2_	10.57 ± 8.18		*T*_0_–*T*_3_	0.067 NS
	*T*_3_	07.71 ± 7.66		*T*_1_–*T*_2_	1.000 NS
				*T*_1_–*T*_3_	0.910 NS
				*T*_2_–*T*_3_	1.000 NS
Group II	*T*_0_	12.31 ± 6.45	0.012*	*T*_0_–*T*_1_	0.676 NS
	*T*_1_	08.85 ± 6.22		*T*_0_–*T*_2_	0.169 NS
	*T*_2_	07.46 ± 5.47		*T*_0_–*T*_3_	0.008**
	*T*_3_	05.00 ± 3.03		*T*_1_–*T*_2_	1.000 NS
				*T*_1_–*T*_3_	0.473 NS
				*T*_2_–*T*_3_	1.000 NS
Group III	*T*_0_	15.50 ± 12.45	0.008**	*T*_0_–*T*_1_	1.000 NS
	*T*_1_	12.28 ± 6.83		*T*_0_–*T*_2_	0.035*
	*T*_2_	06.85 ± 4.91		*T*_0_–*T*_3_	0.018*
	*T*_3_	06.14 ± 5.24		*T*_1_–*T*_2_	0.462 NS
				*T*_1_–*T*_3_	0.277 NS
				*T*_2_–*T*_3_	1.000 NS

*T*_*0*_ prior to application of remineralizing agents, *T*_*1*_ 1 month after the use of remineralizing agents, *T*_*2*_ 3 months after the use of remineralizing agents, *T*_*3*_ 6 months after the use of remineralizing agents, *NS* non-significant

*P < 0.05; **P < 0.01

**Table 3:** Between the group comparisons of visual scores at various time intervals of observation

Time intervals	Groups			Significance (*P* value)
	Group I	Group II	Group III	
	Mean ± SD	Mean ± SD	Mean ± SD	
*T*_0_	15.71 ± 7.94	12.31 ± 6.45	15.50 ± 12.45	0.560 NS
*T*_1_	12.14 ± 8.41	08.85 ± 6.22	12.28 ± 6.83	0.387 NS
*T*_2_	10.57 ± 8.18	07.46 ± 5.47	06.85 ± 4.91	0.416 NS
*T*_3_	07.71 ± 7.66	05.00 ± 3.03	06.14 ± 5.24	0.863 NS

*T*_*0*_ prior to application of remineralizing agents, *T*_*1*_ 1 month after the use of remineralizing agents, *T*_*2*_ 3 months after the use of remineralizing agents, *T*_*3*_ 6 months after the use of remineralizing agents, *NS* non-significant

The changes in the DIAGNOdent scores are described in Table 4. The mean DIAGNOdent score was marginally increased in all groups at the end of *T*_1_ but decreased at the end of *T*_2_ and *T*_3_; the differences were statistically comparable. The changes in the DIAGNOdent scores between the groups at various time intervals of observation (*T*_0_, *T*_1_, *T*_2_, and *T*_3_) are described in Table 5. The mean DIAGNOdent scores in group II and group III were decreased more compared to the decrease in group I; however, the changes were comparable among the three groups (*P* > 0.05).

**Table 4:** Description of DIAGNOdent scores at various time intervals of observation among subjects of three groups

Groups	Time intervals	DIAGNOdent scores	Significance (*P* value)
		Mean ± SD	
Group I	*T*_0_	131.43 ± 41.42	0.744 NS
	*T*_1_	134.00 ± 45.14	
	*T*_2_	121.93 ± 37.31	
	*T*_3_	118.71 ± 46.46	
Group II	*T*_0_	105.54 ± 25.20	0.378 NS
	*T*_1_	107.67 ± 27.73	
	*T*_2_	102.61 ± 34.90	
	*T*_3_	88.85 ± 30.41	
Group III	*T*_0_	119.07 ± 36.27	0.614 NS
	*T*_1_	113.57 ± 40.54	
	*T*_2_	118.78 ± 46.19	
	*T*_3_	100.64 ± 42.33	

*T*_*0*_ prior to application of remineralizing agents, *T*_*1*_ 1 month after the use of remineralizing agents, *T*_*2*_ 3 months after the use of remineralizing agents, *T*_*3*_ 6 months after the use of remineralizing agents, *NS* non-significant

**Table 5:** Between the group comparisons of DIAGNOdent scores at various time intervals of observation

Time intervals	Groups			Significance (*P* value)
	Group I	Group II	Group III	
	Mean ± SD	Mean ± SD	Mean ± SD	
*T*_0_	131.43 ± 41.42	105.54 ± 25.20	119.07 ± 36.27	0.175 NS
*T*_1_	134.00 ± 45.14	107.67 ± 27.73	113.57 ± 40.54	0.900 NS
*T*_2_	121.93 ± 37.31	102.61 ± 34.90	118.78 ± 46.19	0.415 NS
*T*_3_	118.71 ± 46.46	88.85 ± 30.41	100.64 ± 42.33	0.168 NS

*T*_*0*_ prior to application of remineralizing agents, *T*_*1*_ 1 month after the use of remineralizing agents, *T*_*2*_ 3 months after the use of remineralizing agents, *T*_*3*_ 6 months after the use of remineralizing agents, *NS* non-significant

## Discussion

Although the prevention of development of WSLs during comprehensive orthodontic treatment is the goal of every orthodontist, its prevalence is still quite high [21]. Treatment of WSLs present a significant challenge in achieving esthetic excellence [22]. These WSLs can last as long as 5–12 years [7], as natural remineralization through saliva involving mineral gain in the surface layer of WSLs has little improvement on the esthetic and structural properties of the deeper lesions [5]. It therefore becomes important to apply remineralizing agents topically on the tooth surface after debonding to repair the deeper part of the WSLs for better esthetic and structural reinforcement.

The integrity of enamel in the oral cavity under normal conditions is influenced by the dynamic process comprising of alternating periods of demineralization and remineralization. Lot of efforts has been made for reversing the process of demineralization, and fluoride has always remained the gold standard. The presence of trace quantities of fluoride plays a significant role to drive the process in the direction of remineralization [23]. Also, an inverse association between plaque calcium and phosphate levels with enamel demineralization has been found [24]. Thus, efforts have been made to increase the level of fluoride, calcium, and phosphate ions in immediate vicinity of the teeth to enhance the process of remineralization. Various agents like two solution fluoride rinses [25] and tri-calcium phosphate nano-complexes with fluoride ions (Clinpro tooth crème) have been introduced. Milk product-based complexes like CPP-ACP crème have also been tried to deliver calcium and phosphate ions to the tooth surface [4]. Many animal and human experiments also confirmed that CPP-ACP nano-complexes have anticariogenic activity [17, 18, 26]. Thus, the efficacy of fluoride varnish that releases traces of fluoride ions and CPP-ACP plus crème that releases fluoride, calcium, and phosphate ions in immediate vicinity of WSLs were tested in this study.

The DIAGNOdent is a laser fluorescence device used to quantify the enamel demineralization. The main unit generates laser light with a wavelength of 655 nm, which is absorbed by both organic and inorganic material in the tooth and re-emitted as fluorescence within the infrared region. In the presence of caries, fluorescence increases and the change is registered as an increased digital number. The mechanism underlying the enhanced fluorescence in the presence of caries has yet to be established but is presumed to result from the integration of bacterial metabolites rather than crystalline disintegration. The DIAGNOdent readings should always be interpreted with caution because DIAGNOdent readings are often affected by stains, calculus, and plaque [27] and bacterial metabolites [28], which are not directly related to the problems perceived by patients or doctors. Therefore, the combined use of technology-based methods and visual assessment is the best approach for evaluating the WSLs.

We observed that the use of 1000 ppm of fluoride toothpaste twice daily alone had beneficial effect on the regression of WSLs. Similar to our observation, Hoffman et al. [29] also observed that the use of traditional fluoride toothpaste alone was effective in improving white spot lesions during orthodontic treatment. However, in contrast to our observation, many previous studies [13, 16] reported that the toothpaste alone was not effective in reducing the post-orthodontic WSLs. Zantiner et al. [12] also observed that the use of either sodium fluoride toothpaste (1500 ppm) or toothpaste-containing amine fluoride (1250 ppm) for 6 months had no effect on the WSLs. However, Alexander et al. [16] reported that brushing twice daily with 5000-ppm fluoride toothpaste was more effective in the reversal of enamel demineralization than tooth brushing with a 1000 ppm of fluoride toothpaste. The concentration and doses of fluoride application are controversial. Fluoride agents that release a high dose of fluoride initially (burst effect) are more effective for increasing enamel resistance against decalcification [30]. A low concentration of fluoride is more effective in enamel remineralization [31]. The high dose of fluoride physically blocks the surface layer of enamel to penetration of calcium ions to subsurface layers [32]. Thus, high dose of fluoride is recommended in inhibiting lesion formation and low dose of fluoride for the remineralization and controlling lesion progression.

The results of the present study however revealed that brushing twice daily with 1000 ppm of fluoride toothpaste along with 5 % NaF varnish or CPP-ACP plus crème was more effective in remineralizing the WSLs. But their efficacy was comparable to the remineralization efficacy of fluoride toothpaste alone at various intervals of observations. When fluoride varnish is applied to the tooth surface, it forms a reservoir of fluoride ions which get released slowly and thus continuously reacted with the hydroxy-apatite crystals of enamel over a long period of time leading to deeper penetration and formation of fluoro-hydroxyapatite crystals. Similar to our observation, many authors also observed reversal in the enamel lesions following application of fluoride varnish [14, 16]. Boyd [14] found that twice daily use of 1100-ppm fluoride toothpaste along with 0.05 % NaF rinse or topical application of 0.4 % SnF_2_ gel had additional protection against enamel decalcification. Feagin [33] reported that fluoride increased the rate of calcium phosphate deposition during remineralization of acid-softened enamel and itself got incorporated into the mineral thus formed. Brushing twice daily with 1000-ppm fluoride toothpaste results in the formation of CaF_2_ and also deposition of fluoride in the superficial layers of enamel, in contrast to the fluoride varnish in which the slow release of fluoride ions results in the formation of more stable fluoro-hydroxyapatite compound [34].

The present study showed better remineralization of WSLs following the use of CPP-ACP plus crème along with 1000 ppm of fluoride toothpaste. But between the group comparison showed no additional remineralization efficacy of CPP-ACP plus crème when used with 1000 ppm of fluoride toothpaste. However, literature supports that application of CPP-ACP crème along with fluoride toothpaste on daily basis had better remineralization potential than once professional application of fluoride varnish and twice daily use of fluoride toothpaste [35, 36]. The synergistic effect of CPP-ACP and fluoride in reducing the WSLs is due to the formation of CPP-stabilized amorphous calcium fluoride phosphate, resulting in increased concentration of bioavailable calcium and phosphate ions [37]. The CPP-bound ACP acts as a reservoir of calcium phosphate ions, including the neutral ion pair CaHPO_4_ which is formed in the presence of acid [38]. When acid is formed by the plaque bacteria, the CPP-bound ACP buffers the plaque pH, and in doing so, it dissociates to calcium and phosphate ions including CaHPO_4_. The increased plaque calcium and phosphate ions offset any fall in pH, thereby preventing enamel demineralization. In the presence of fluorides, formation of CPP-ACFP nano-complexes takes place, and when the pH falls, breakage of the nano-complex leads to formation of calcium ions, phosphate ions, and neutral species CaHPO_4_ and HF. These ions following concentration gradient move inside the subsurface lesion, thus leading to formation of fluorapatite. Feagin [33] observed that in the presence of fluoride, the rate of calcium and phosphate deposition increased during remineralization and accelerated the rate of enamel surface rehardening. Similarly, Koulourides et al. [39] also found a relative increase in the rate of calcium and phosphate deposition from solutions containing fluoride. Akin et al. [40] found 58 % reduction in the WSLs following 6 months of use of CPP-ACP crème twice daily along with fluoride toothpaste. Andersson et al. [41] observed that daily topical application of a dental crème containing CPP-ACP for 3 months followed by a 3-month period of daily tooth brushing with fluoridated toothpaste helped in the complete elimination of the post-orthodontic WSLs. Llena et al. [17] observed that 4-week use of CPP-ACFP was superior to duraphate fluoride varnish in remineralizing smooth surface WSLs. However, Beerns et al. [18] observed that the use of CPP-ACFP crème for 12 weeks had no clinical advantage over normal hygiene in the remineralization of WSLs.

Although we observed that the additional use of fluoride varnish and CPP-ACP plus crème to daily use of fluoride toothpaste was more effective in reducing the severity of WSLs, this effectiveness was comparable with the remineralization efficacy of fluoride toothpaste alone. Thus, orthodontists can routinely prescribe traditional fluoride toothpastes for the management of post-orthodontic WSLs. However, more similar studies are required to confirm the present findings. The limitation of the present study was that the visual and DIAGNOdent scores of each tooth were added to find out a mean score for a subject. However, for better results, the mean visual and DIAGNOdent scores for each individual tooth would have been calculated separately and considered for statistical analysis.

## Conclusions

1. The use of 1000 ppm of fluoride toothpaste twice daily was effective in the remineralization of post-orthodontic WSLs.
2. The use of 5 % NaF varnish in addition to twice daily use of 1000 ppm of fluoride toothpaste had no additional beneficial effect in the remineralization of post-orthodontic WSLs.
3. Twice daily use of CPP-ACP plus crème along with twice daily use of 1000 ppm of fluoride toothpaste had no added benefit in the remineralization of post-orthodontic WSLs.
